# Transcriptional Profiling of Host Gene Expression in Chicken Embryo Fibroblasts Infected with Reticuloendotheliosis Virus Strain HA1101

**DOI:** 10.1371/journal.pone.0126992

**Published:** 2015-05-14

**Authors:** Ji Miao, Yanqing Bao, Jianqiang Ye, Hongxia Shao, Kun Qian, Aijian Qin

**Affiliations:** 1 Ministry of Education Key Laboratory for Avian Preventive Medicine, Yangzhou University, Yangzhou, Jiangsu Province, China; 2 Key Laboratory of Jiangsu Preventive Veterinary Medicine, Yangzhou University, Yangzhou, Jiangsu Province, China; 3 Jiangsu Co-innovation Center for Prevention and Control of Important Animal Infectious Diseases and Zoonoses, Yangzhou University, Yangzhou, Jiangsu Province, China; Public Health Research Institute at RBHS, UNITED STATES

## Abstract

Reticuloendotheliosis virus (REV), a member of the Gammaretrovirus genus in the Retroviridae family, causes an immunosuppressive, oncogenic and runting-stunting syndrome in multiple avian hosts. To better understand the host interactions at the transcriptional level, microarray data analysis was performed in chicken embryo fibroblast cells at 1, 3, 5, and 7 days after infection with REV. This study identified 1,785 differentially expressed genes that were classified into several functional groups including signal transduction, immune response, biological adhesion and endocytosis. Significant differences were mainly observed in the expression of genes involved in the immune response, especially during the later post-infection time points. These results revealed that differentially expressed genes *IL6*, *STAT1*, *MyD88*, *TLRs*, *NF-κB*, *IRF-7*, and *ISGs* play important roles in the pathogenicity of REV infection. Our study is the first to use microarray analysis to investigate REV, and these findings provide insights into the underlying mechanisms of the host antiviral response and the molecular basis of viral pathogenesis.

## Introduction

Reticuloendotheliosis virus (REV) is classified as a member of the genus Gammaretrovirus in the family Retroviridae and causes an immunosuppressive, oncogenic and runting-stunting syndrome in multiple avian hosts[1]. REVs comprise a variety of strains, including nondefective REV-A, defective REV-T, spleen necrosis virus (SNV), chick syncytial virus (CSV), and duck infectious anaemia virus (DIAV)[2]. Recently, the co-infection of REV with other avian viruses has been reported, potentially representing additional dangers to the poultry industry[3, 4]; moreover, the risks associated with the worldwide distribution of REVs are unknown[5–7]. The enhancement of avian reticuloendotheliosis disease due to concomitant infection is most likely a consequence of its immunosuppressive ability [8–10]. However, the mechanism of REV-induced tumourigenesis and immunosuppression has not yet been fully characterised.

With the rapid development of microarray technology, an increasing number of veterinary medicine studies have investigated host gene transcriptional responses to infection by various avian viruses[11–14]. REV, avian leucosis virus (ALV), and Marek’s disease virus (MDV) are the main causes of neoplastic diseases in avian hosts. Recently, our group reported the expression kinetics of transcripts and their relative expression profiles for both MDV infection and ALV-J infection[13, 15]. To the best of our knowledge, the effects of REV on changes in global gene expression in infected host cells have not been previously reported. Thus, the objective of this study was to investigate the transcriptional profile of host responses to REV infection at different time points post-infection in chicken embryo fibroblast cells using microarray analysis.

In this study, we analysed changes in the expression of cellular genes in chicken embryo fibroblasts (CEFs) infected with the REV HA1101 strain using microarray analysis. A total of 1,785 differentially expressed genes were identified. Analyses and functional studies of these genes and the relevant signalling pathways may provide novel information that will increase our understanding of the pathogenesis of REV and the mechanisms of in-vitro host responses over time.

## Materials and Methods

### Virus infection assay

Reticuloendotheliosis virus strain HA1101 (GenBank accession number: KF305089.1) was isolated from commercial layer chickens in Jiangsu, China, and stored at the Key Laboratory of Jiangsu Preventive Veterinary Medicine. The virus was propagated on a monolayer of primary CEFs prepared from 10-day-old specific-pathogen free (SPF) chicken embryos (Merial Vital Laboratory Animal Technology, China). In this study, CEFs were plated at a density of 1×10^4^ cells per well in 24-well culture plates and then inoculated with pre-treated virus suspensions. The CEFs were infected with REV at a multiplicity of infection (MOI) of 1. After a 2 h exposure to virus, the cells were washed three times and cultured in Dulbecco’s modified Eagle medium (DMEM; GIBCO, China) supplemented with 1% foetal bovine serum (FBS; GIBCO, China) at 37°C in a 5% CO_2_ atmosphere. REV infection was verified using an indirect immunofluorescence assay with a mouse anti-REV monoclonal antibody[16]. All cell cultures were seeded simultaneously. Cells were harvested at 1, 3, 5, and 7 days post-infection (dpi). All animal experiments were conducted in accordance with the guidelines provided by the Chinese Council on Animal Care. All experiments complied with institutional animal care guidelines and were approved by the University of Yangzhou Animal Care Committee.

### RNA isolation and array hybridisation

Cellular and viral RNAs were extracted using the AxyPrep Multisource Total RNA Miniprep Kit (AXYGEN, China) according to the manufacturer’s protocol. Sample RNAs were quantified using a spectrophotometer and maintained at -70°C for future use. For the microarray analysis, RNA quality was assessed using an Agilent Bioanalyzer (Agilent Technologies, USA). Sample RNA integrity numbers (RINs) were obtained to assign values to RNA measurements in an unambiguous manner. Total RNAs were reverse transcribed to produce double-stranded cDNA, from which cRNAs were synthesised and then labelled with cyanine-3-CTP. The labelled cRNAs were hybridised onto Agilent Chicken Gene Expression (4*44K, Design ID: 026441) microarrays[17]. After washing, the arrays were scanned using an Agilent Scanner G2505C (Agilent Technologies, US). The sample labelling, microarray hybridisation and washing were performed based on standard protocols provided by the Shanghai Oebiotech Corporation.

### Microarray data analysis

To analyse array images, raw data were extracted using Feature Extraction software (version10.7.1.1, Agilent Technologies) and then analysed and normalised using the quantile algorithm. Genespring (version11.0 Agilent Technologies, US) was employed to perform a basic analysis of the raw data. The microarray data have been submitted to the GEO database (http://www.ncbi.nlm.nih.gov/geo/) under accession number GSE66320. Differentially expressed genes were then identified based on fold-changes and P-values calculated using Student's t-test. The threshold for up- and down-regulated genes was a fold change > = 2.0 and a P value < = 0.05. Subsequently, Gene Ontology (GO) and Kyoto Encyclopaedia of Genes and Genomes database (KEGG) (CapitalBio, Beijing China) analyses were applied to determine the potential roles of these differentially expressed mRNAs based on GO terms or pathways. Finally, hierarchical clustering was performed by using GeneSpring software to visualise the distinguishable gene expression patterns among samples[15]. All presented data represent averaged changes in gene expression for 3 independent replicates.

### Quantitative reverse transcription PCR (qRT-PCR)

RNA isolated from the CEFs of each participant was reverse-transcribed into cDNA using the PrimeScript RT reagent kit with gDNA Eraser (Takara, China). qRT-PCR targeting of selected cellular genes was performed using the SYBR Premix Ex Taq II (TaKaRa, China) kit with a 7500 Real-time PCR System (Applied Biosystems, USA). The optimal primers were synthesised by Invitrogen (Shanghai, China), and their sequences are listed in Table 1. Chicken 18S ribosomal RNA was targeted to analyse relative gene expression using the Livak and Schmittgen 2^-ΔΔCT^ method[18]. As 18S rRNA is a reliable normalisation gene for real time PCR[19], we used this housekeeping gene evaluated in this study. All samples were run in duplicate to guarantee the reproducibility of the amplification.

**Table 1:** Primer sequences for qRT-PCR.

Gene symbol	Forward primer (5'-3')	Reverse primer (5'-3')
*OASL*	GAGATAGAGAAGGAGTGGTG	GTAGACTGTGGTCTTGTTAC
*IL6*	GTGATAAATCCCGATGAAGT	GTCTTCTCCATAAACGAAGT
*CCL19*	AGGTATTTGCTGCTAGATGT	GCCTATGGGCTTTTATTTTTATT
*CCL20*	AAGATTGCTGTCTGTCTTAC	CTTCCTTAGGATTTACGCAG
*TLR3*	GATTGCAGTCTCAGTACATT	AACATGAATTGCATCACAAC
*TLR4*	ATTCAATGATATGCCACACA	TGAGGAATAGAAACACTCCT
*RSAD2*	GAGAACCATTTCTTCAGGAC	TCACCATACTTCTTGAACCA
*MX1*	AATAAGGCTACTATCCCACA	GTGTACTTTTGGAGTTCCTT
*ISG12-2*	GGAATTGCAAGAGGTTCTC	CCCATTTCTTGTAGAGTAGC
*STAT1*	AAGTTTTTGGAGCAAGTTCA	AGCACTGTAGCAAAAGATAC
*SOCS1*	GGAGACCTTTGATTGTCTTT	TCTCTTCCAAAAGTCTTCAC
*18S rRNA*	TCAGATACCGTCGTAGTTC	TCCGTCAATTCCTTTAAGTT

## Results

### REV infection of CEFs

**Fig 1:**
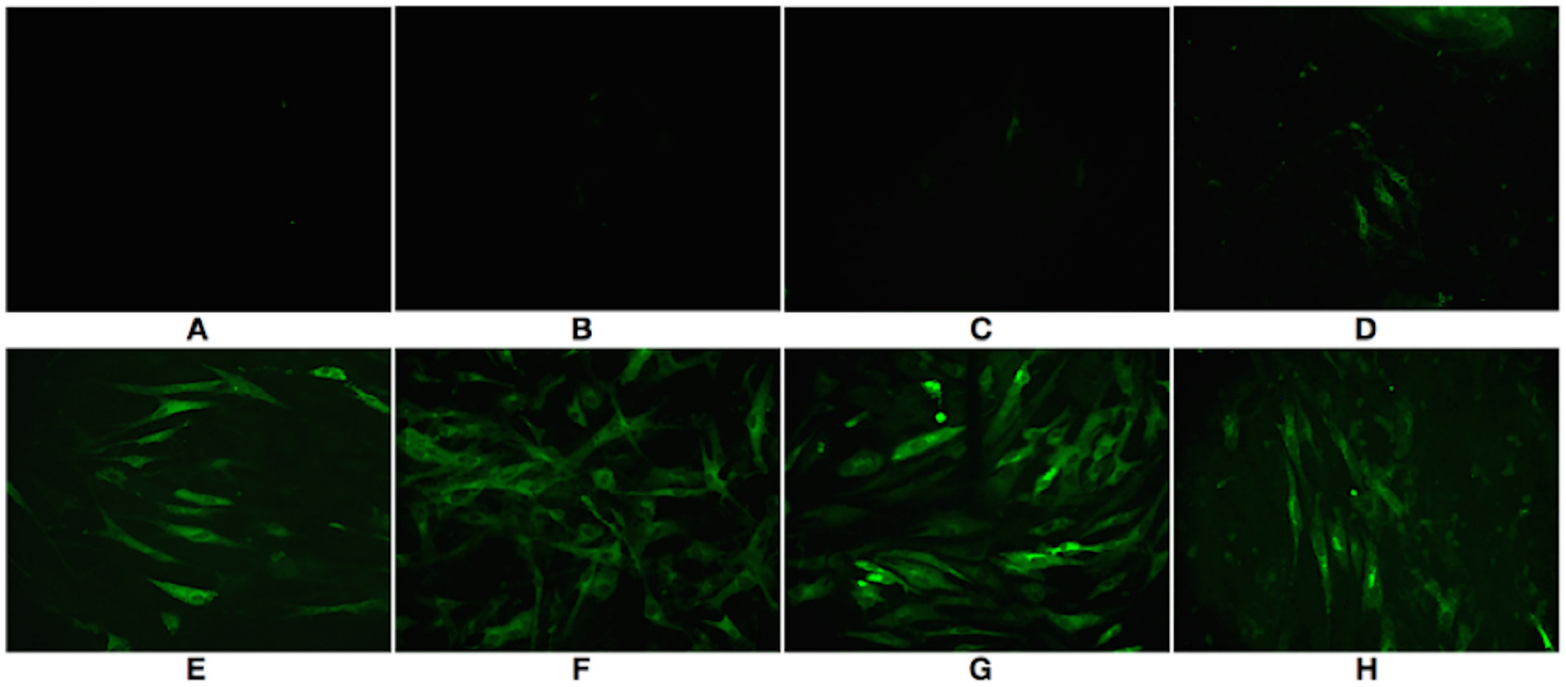
REV infection in chicken embryo fibroblasts. Detection of REV group antigen in infected monolayer of CEFs was visualised by IFA on day 1 (A), day 2 (B), day 3 (C), day 4 (D), day 5 (E), day 6 (F), day 7 (G) and day 10 (H) using fluorescence microscopy at an original magnification of 400×.

Viral infection was confirmed by detecting the REV group antigen using IFA (Fig 1), as REV does not induce cytopathologic effects (CPE) in CEFs[20]. The results showed that weak fluorescence could be observed beginning at 3 days post-infection. Subsequently, the fluorescence signal became more intense and was brightest on day 7 post-infection. Several days thereafter, both infected and uninfected CEFs were observed to be uneven in shape with poor proliferative activity.

### Significant transcripts/genes and clustering

In this investigation, we initially performed gene expression analysis using Agilent’s Chicken Gene Expression microarrays containing 43,803 probes. We then applied Student's t-test and fold-change cutoff for the selection of highly significant transcripts in infected CEFs compared with the control samples at 1, 3, 5 and 7 dpi. We identified a total of 3,791 differentilly changed transcripts. Among these, 689, 1,196, 1,699, and 2,676 transcripts were differentially regulated with at least two-fold differences relative to uninfected cells at 1, 3, 5, and 7 dpi, respectively. Excluding transcripts with undefined functions in the GO database (accessed December 25, 2014) and multiple probes for the same gene, we identified 1,785 differentilly changed genes with significant expression levels. The number of differentially expressed genes at each time point is listed in Table 2.

**Table 2:** The numbers of significant differentially expressed transcripts and genes.

Time-points	Differentially expressed transcripts	Up-regulated transcripts	Down-regulated transcripts	Differentially expressed genes
1 Day	689	413	276	346
3 Day	1,196	743	453	613
5 Day	1,699	1,036	663	851
7 Day	2,676	1,480	1,196	1,333

**Fig 2:**
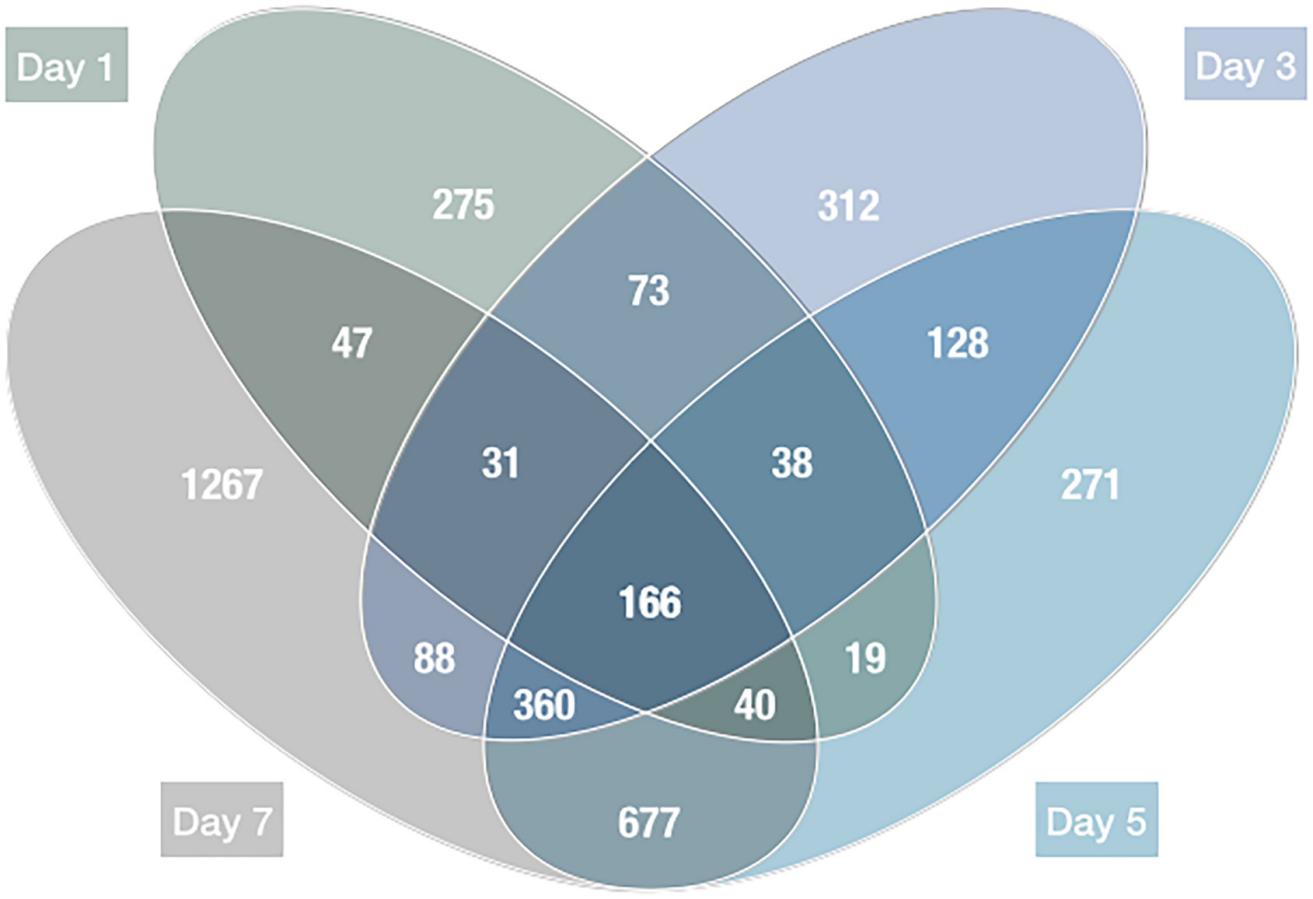
Venn diagram of significantly differentially expressed transcripts over the time course of REV infection. The image displays the number and overlap of differentially expressed transcripts in response to in vitro REV infection at 1, 3, 5, and 7 days post-infection. The numbers of transcripts differentially expressed at more than one time point are shown in the overlapping regions. Additionally, the intersection of the four circles indicates transcripts that were up- or down-regulated at all time points of REV infection.

**Fig 3:**
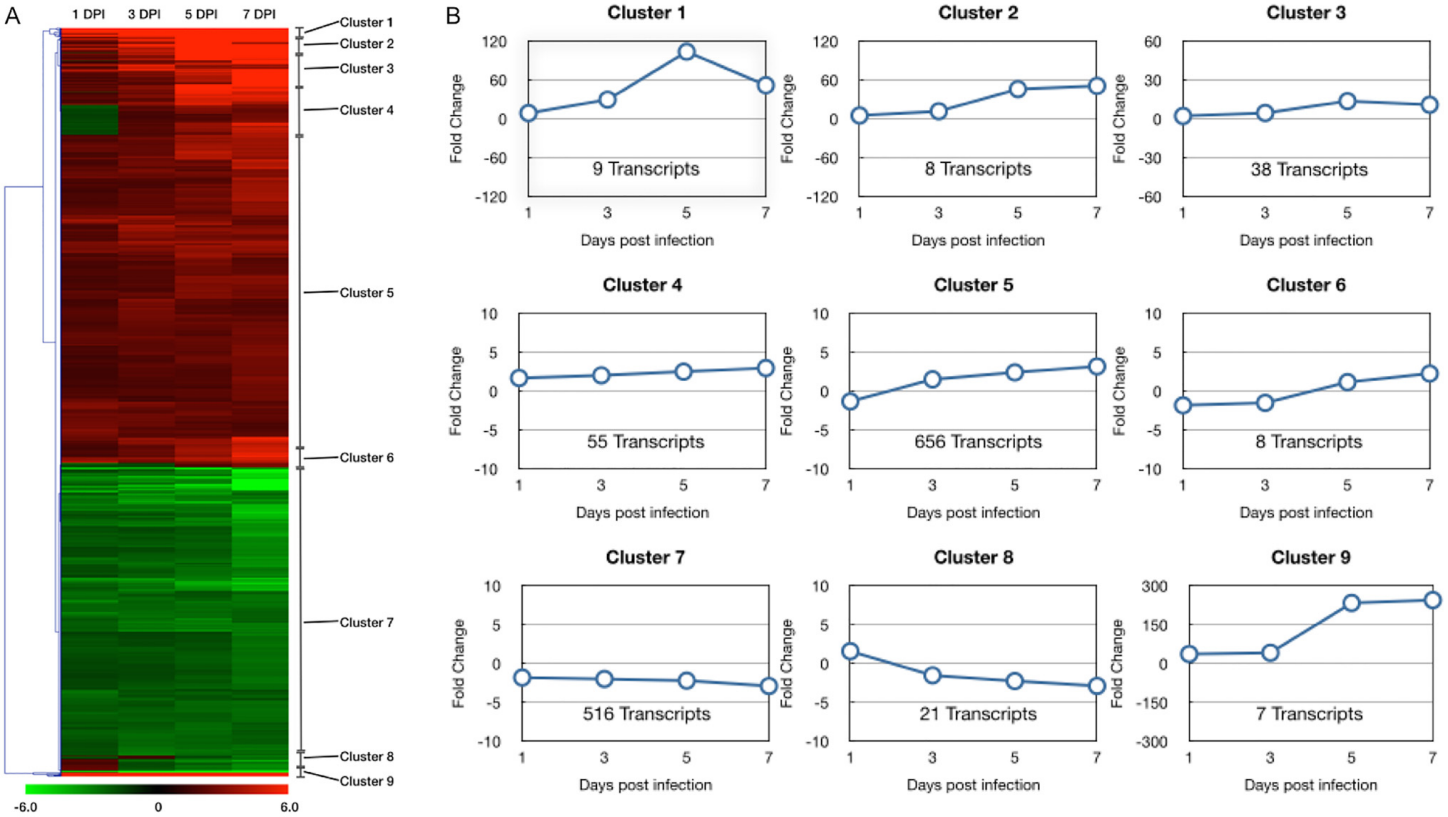
Hierarchical clustering (A) and k-means clustering (B) of differentially expressed transcripts of REV infected CEFs at different post-infection time points. Expression profiles of differentially expressed transcripts with p < 0.05 at all time points and fold changes > +/- 2 at one or more time points. These significantly regulated transcripts were clustered into 9 distinct groups having similar expression response profiles over the time course of REV infection.

Additionally, Venn diagram analysis revealed that 166 transcripts were represented by differentially expressed transcripts at all four tested time points (Fig 2). Of these transcripts, 77 genes were identified and sorted by data analysis based on the highest standard deviations using the mean values of all time points (S1 Table). Hierarchical and K-means clustering resulted in the identification of 9 distinct patterns of transcript variations at the four different time-points (Fig 3). Thus, expression pattern of most differentially expressed transcripts was similar and formed in two clusters, Cluster 5 and Cluster 7 in Fig 3. It showed these gradual changes during the time course of REV infection, and a complete list of significantly changed transcripts in each cluster was shown in S2 Table.

### GO analysis of differentially expressed genes

**Fig 4:**
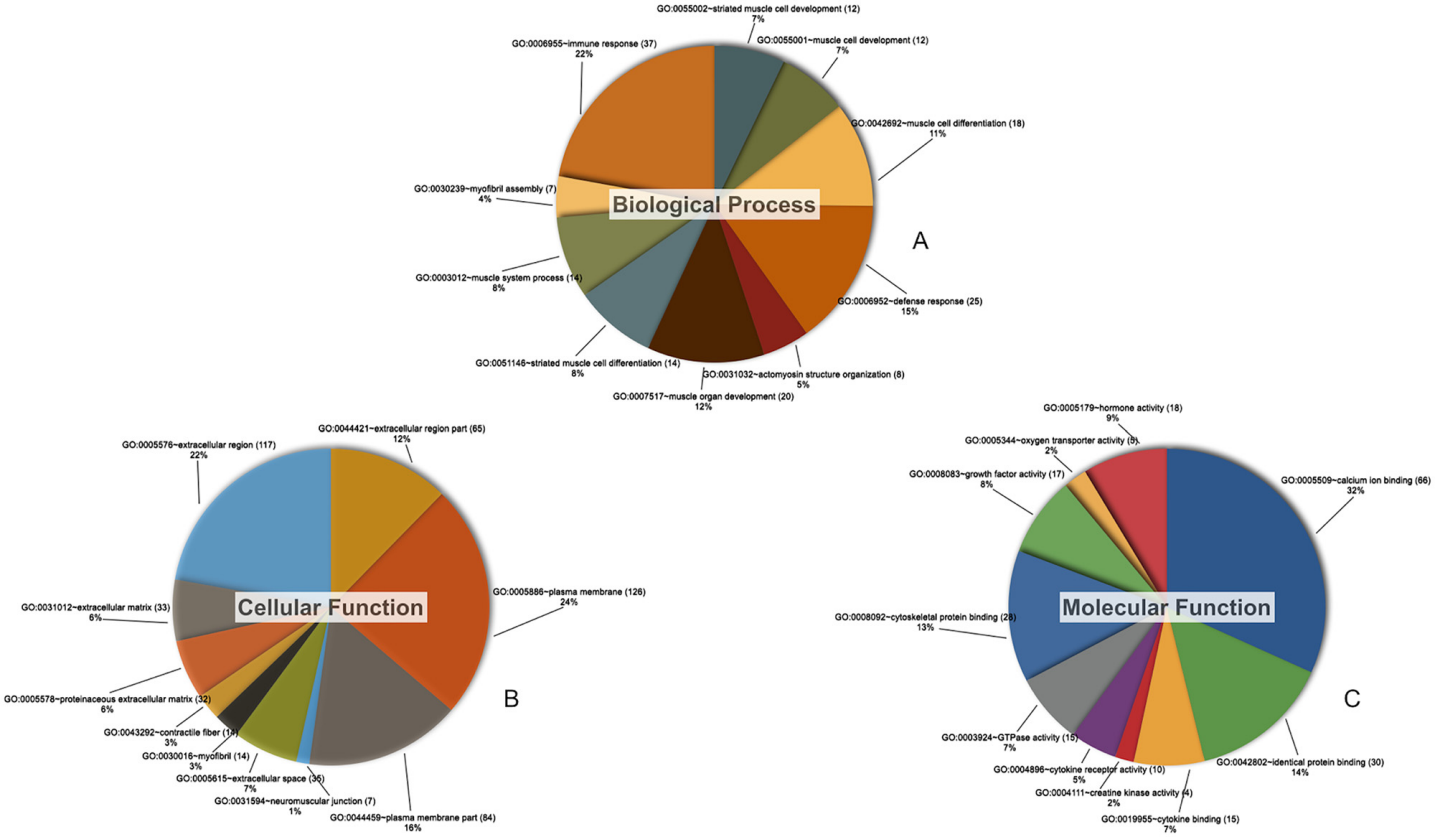
Gene ontology analysis of differentially expressed genes according to their biological process (A), cellular function (B), and molecular function (C). Each colour represents a different GO term, and the number of enriched target genes are shown after the name of the GO term. Only the top ten GO terms in each category are listed here. The complete GO analysis dataset is shown in S3 Table.

For all four time points, Gene Ontology (GO) category analysis of the genes corresponding to the 1,785 differentially expressed transcripts was performed using the web-based bioinformatics tool Database for Annotation, Visualisation, and Integrated Discovery (DAVID, http://david.abcc.ncifcrf.gov/). Functional analysis revealed that 139 GO terms in the biological process category, 23 GO terms in the cellular component category, and 24 GO terms in the molecular functions category were found to be significantly enriched (P < 0.05). These results provide an overview of the host response to REV infection with respect to the top 10 enriched GO terms of differentially expressed genes in each category (Fig 4). The following GO terms were most commonly implicated in the biological process category: immune response, biological adhesion, regulation of phosphorylation, antigen processing and presentation, defence response, cell surface receptor linked signal transduction, regulation of cell proliferation, defence response, and response to organic substance. Additionally, we identified a total of 37 differentially expressed genes associated with the immune response term during the time course of REV infection (S4 Table).

### KEGG pathway analysis of differentially expressed genes

To explore the various biological processes involved in REV infection, the differentially expressed genes were mapped into canonical signalling pathways using KEGG analysis. KEGG data analysis revealed that the differentially expressed genes were enriched in 8 pathways (P < 0.05) (Table 3). This finding suggests that the viruses and host cells utilise different strategies that may be associated with the pathogenesis induced by REV infection. Differentially expressed genes involved in certain critical signalling pathways during REV infection are shown in Table 4.

**Table 3:** KEGG pathway analyses in response to REV infection.

KEGG Pathways	Gene Count	P-Value	Genes
gga04060:Cytokine-cytokine receptor interaction	31	1.16E-03	*TNFRSF6B*, *LOC422316*, *IL1R2*, *IL22RA1*, *OSMR*, *TNFSF15*, *KITLG*, *TNFRSF8*, *CX3CL1*, *CCL5*, *CXCL12*, *IFNA3*, *TNFRSF11B*, *CXCR4*, *IL10RA*, *TNFRSF19*, *CSF3R*, *IL2RG*, *GHR*, *CSF1R*, *IL6*, *TNFSF4*, *FLT1*, *TNFRSF13B*, *LIFR*, *HGF*, *IL6RA*, *TNFSF10*, *CNTF*, *PRLR*, *PDGFRA*
gga04514:Cell adhesion molecules (CAMs)	22	4.29E-03	*PTPRC*, *SELP*, *BLB1*, *CLDN5*, *ITGB2*, *CDH1*, *ITGB1*, *CDH4*, *PDCD1*, *CDH5*, *NRCAM*, *NCAM1*, *ALCAM*, *VCAM1*, *ITGA9*, *SDC1*, *CD80*, *BF2*, *BF1*, *MR1*, *B-MA2*, *ICOSLG*
gga04512:ECM-receptor interaction	17	9.94E-03	*TNC*, *COL3A1*, *HSPG2*, *COL2A1*, *VTN*, *ITGB3*, *ITGB1*, *GP9*, *HMMR*, *ITGA9*, *SDC1*, *CD36*, *COL6A3*, *GP1BA*, *SV2C*, *FN1*, *THBS4*
gga03320:PPAR signalling pathway	15	5.33E-03	*ACOX2*, *PPARG*, *FADS2*, *CPT1A*, *MMP1*, *CD36*, *APOA1*, *ACSL1*, *FABP3*, *SLC27A6*, *FABP4*, *GK*, *SCD5*, *FABP7*, *PLTP*
gga03010:Ribosome	15	4.54E-02	*RPSA*, *RPL35A*, *RPLP2*, *RPS28*, *RPS16*, *RPL7*, *RPLP0*, *RPS14*, *RPL8*, *RPL3*, *RPS12*, *RPL3L*, *RPL5*, *RPL7A*, *RPL4*
gga04672:Intestinal immune network for IgA production	9	3.05E-02	*IL6*, *BLB1*, *CD80*, *CXCR4*, *TNFRSF13B*, *TGFB3*, *B-MA2*, *CXCL12*, *ICOSLG*
gga02010:ABC transporters	9	3.67E-02	*LOC420606*, *TAP2*, *TAP1*, *ABCC3*, *ABCC2*, *ABCB6*, *ABCA3*, *ABCG1*, *ABCC6*
gga04623:Cytosolic DNA-sensing pathway	8	4.00E-02	*IFNA3*, *TMEM173*, *IL6*, *IRF7*, *NFKBIA*, *NFKB1*, *CCL5*, *ADAR*

**Table 4:** Differentially expressed genes in REV infection involved in signalling pathways.

KGEE Pathways	Gene Count	P-Value	Genes
gga04010:MAPK signaling pathway	27	4.99E-01	*FGFR2*, *TRAF2*, *IL1R2*, *FGFR3*, *FGF14*, *FGF16*, *TGFB3*, *FGF10*, *NFKB1*, *FGF13*, *FGF12*, *NFKB2*, *MAP3K4*, *RAC2*, *PAK1*, *PRKACB*, *AKT3*, *PTPN5*, *CACNG4*, *CACNG3*, *CACNA2D3*, *CACNG1*, *DUSP4*, *DUSP1*, *PDGFRA*, *RAP1B*, *CACNA1D*
gga04144:Endocytosis	23	3.32E-01	*FAM125B*, *FGFR2*, *LOC422316*, *PARD6B*, *FGFR3*, *FLT1*, *ERBB4*, *PIP5K1A*, *SRC*, *ADRB2*, *RAB31*, *TFRC*, *PSD*, *CXCR4*, *GRK6*, *PDGFRA*, *BF2*, *GRK7*, *BF1*, *IL2RG*, *GRK5*, *MR1*, *CSF1R*
gga04630:Jak-STAT signaling pathway	19	1.41E-01	*PTPN6*, *IL6*, *IL22RA1*, *OSMR*, *SOCS3*, *SOCS1*, *LIFR*, *STAT1*, *IL6RA*, *IFNA3*, *SPRY1*, *CNTF*, *PRLR*, *IL10RA*, *CSF3R*, *IL2RG*, *AKT3*, *GHR*, *IL13RA2*
gga04310:Wnt signaling pathway	15	6.89E-01	*WNT5B*, *MMP7*, *FZD3*, *DKK2*, *WNT4*, *RAC2*, *SFRP2*, *SFRP4*, *WNT11*, *PRKACB*, *WNT9A*, *SOX17*, *PLCB1*, *PLCB2*, *WNT8A*
gga04620:Toll-like receptor signaling pathway	14	1.33E-01	*IL6*, *LY96*, *TLR3*, *NFKBIA*, *NFKB1*, *TLR4*, *CCL5*, *STAT1*, *TLR2-1*, *IFNA3*, *TLR2-2*, *CD80*, *IRF7*, *AKT3*
gga04210:Apoptosis	8	7.86E-01	*TRAF2*, *PRKAR2B*, *TNFSF10*, *NFKBIA*, *NFKB1*, *PRKACB*, *ATM*, *AKT3*
gga04621:NOD-like receptor signaling pathway	7	3.58E-01	*IL6*, *CARD9*, *HSP90AA1*, *PSTPIP1*, *NFKBIA*, *NFKB1*, *CCL5*
gga04622:RIG-I-like receptor signaling pathway	7	5.01E-01	*IFNA3*, *TRAF2*, *TMEM173*, *IFIH1*, *IRF7*, *NFKBIA*, *NFKB1*

### Verification of the microarray results by qRT-PCR

To confirm the results of the microarray analysis at different time points after REV infection, total RNA was extracted from CEFs and analysed using real-time RT-PCR. As shown in Table 5, we selected 11 genes including *STAT1*, *ISG12-2*, *TLR-3*, *IL-6*, and *SOCS1* and quantified their expression by qRT-PCR. The overall results generally matched the microarray data, indicating the reliability of the microarray analysis. Although variations were observed between these two analyses, these were most likely due to differences in intrinsic features between the techniques and procedures. Thus, the qRT-PCR results showed the same relative regulation of expression patterns as those observed by the microarray analysis, and the microarray data were dependable indicators of variations in gene expression.

**Table 5:** Comparison of fold changes obtained using microarray and qRT-PCR analysis.

Gene Symbol	Day 1		Day 3		Day 5		Day 7	
	MA	qRT-PCR±SD	MA	qRT-PCR±SD	MA	qRT-PCR±SD	MA	qRT-PCR±SD
*OASL*	7.40	8.88 ± 1.03	35.20	22.5 ± 3.88	109.80	67.66 ± 10.34	56.10	32.81 ± 5.65
*IL6*	-1.24	1.03 ± 0.12	-1.03	-1.10 ± 0.14	1.77	-1.26 ± 0.18	10.45	10.15 ± 1.22
*CCL19*	1.67	2.80 ± 0.30	5.81	9.31 ± 1.12	24.32	24.03 ± 3.07	39.91	58.42 ± 7.72
*CCL20*	1.06	1.47 ± 0.18	2.29	2.02 ± 0.23	2.95	1.17 ± 0.10	16.69	18.83 ± 2.09
*TLR3*	1.99	2.52 ± 0.28	6.26	5.53 ± 0.46	19.46	15.91 ± 0.83	25.06	12.11 ± 1.39
*TLR4*	-1.26	1.11 ± 0.29	-3.79	-3.23 ± 0.29	-3.21	-2.37 ± 0.24	-3.06	-4.76 ± 1.40
*RSAD2*	29.90	38.32 ± 4.46	75.70	66.98 ± 4.51	334.70	319.8 ± 13.81	361.40	122.98 ± 18.35
*MX1*	12.50	29.68 ± 8.37	28.70	42.11 ± 2.44	217.60	314.52 ± 43.74	204.20	150.82 ± 23.89
*ISG12-2*	33.40	77.15 ± 10.48	51.60	53.23 ± 3.04	252.70	230.43 ± 16.51	253.10	235.57 ± 26.92
*STAT1*	2.00	2.12 ± 0.23	3.00	2.47 ± 0.27	8.60	7.86 ± 0.56	7.10	6.80 ± 0.81
*SOCS1*	1.34	1.24 ± 0.22	1.66	1.46 ± 0.13	4.54	2.95 ± 0.40	5.05	4.68 ± 0.52

The gene expression levels of 11 genes at four different time points in microarray analysis were confirmed by qRT-PCR. “-” Indicates gene was downregulated; without “-” Indicates gene was upregulated. MA: Microarray, SD: Standard Deviation.

## Discussion

Microarray data analysis is a core technology in transcriptomics and is widely used in life sciences research. This type of analysis has also made invaluable contributions to research in chickens[21]. We first used a transcriptomics approach involving microarrays to study the molecular profile of virus-infected cells and obtain a dynamic overview of the altered gene expression in CEFs responding to REV infection.

The number of differentially expressed genes from chicken embryo fibroblasts infected with REV increased dramatically until 7 days post-infection. This result is consistent with that observed in chicken embryo lung cells infected with ILTV at 0, 1, 3, 5, and 7 dpi, in which 789 differentially expressed genes involved in the immune system, cell cycle regulation, matrix metalloproteinases and cellular metabolism were identified[22]. In addition to previously reported host genes expressions following infection with other avian viruses, we identified changes in the expression of both conserved and unique genes by a comparative analysis of REV-regulated gene. In this study, the host cells responded vigorously to the replication of the virus, resulting in the differential regulation of many cellular genes over time. Therefore, there was an increase in the number of significantly altered host genes during the time course of infection.

We found that some of the differentially expressed genes were involved in cytokine-cytokine receptor interactions, metabolic processes, cell adhesion, and immune responses. We also identified differentially expressed antiviral and immunosuppressive genes and pathways associated with the pathogenesis of REV infection.

### Host immune system response to REV infection

We observed various changes in the infected cells, including altered regulation of the expression of a total of 37 genes associated with the immune response. Of these differentially expressed genes, REV infection may have resulted in the strong induction of IFN-stimulated genes (ISG) such as *RSAD2*, *ISG12-2*, *OASL*, *MX1*, and *IFIT5* to ensure viral survival. This phenomenon is consistent with human retrovirus[23] and avian retrovirus infections[15]. Classical ISGs have been increasingly studied and characterised in chickens (e.g., the IFN signal transduction cascade, the Myxovirus resistance proteins (Mx), Protein kinase R (PKR), and 2’-5’-oli-goadenylate synthetase (OAS))[24]. In this study, the differential expression of ISGs was higher during the late stage when the cells were infected with a much larger dose of virus. Indeed, at day 1 post-infection, some of the genes displayed no differences in expression levels. ISGs have continued to be identified and their antiviral activities have been characterised because these clearly diverse factors are critically important for viral pathogenesis[25]. Our current understanding of how ISGs display multiple antiviral functions is largely derived from studies involving the interference of various steps of the viral life cycle[26]. Based on these studies, the antiviral abilities of ISGs are due to the collaboration of multiple ISGs at steps ranging from virus penetration through virus release rather than the function of any single ISG[27].

The induction of the antiviral innate immune response depends on the recognition of pathogen-associated molecular patterns (PAMPs) of viral components by pattern recognition receptors. Members of the Toll-like receptor (TLR) family have emerged as key sensors that recognise viral components such as nucleic acids[28, 29]. In this study, the transcriptional level of TLR-3 molecules was significantly increased, whereas the transcriptional level of TLR-2 and TLR-4 was decreased after REV infection. TLR-3 is known to play a key role in the host response to virus infection by detecting virus-derived dsRNA in intracellular vesicles[30], whereas TLR-2 and TLR-4 recognise viral structural proteins on the plasma membrane [31]. Our results suggested that the expression of TLR-2 and TLR-4 might be inhibited, causing a diminished immune response to REV infection. In birds, recognition triggers the downstream signal transduction to activate NF- kappaB or IRF-3/7 and finally induces interferon and inflammatory cytokine production[32]. Thus, the up-regulation of STAT-1 could be due to increases in IRF-7-mediated signalling. In this study, differential expression of STAT-1 was also associated with higher cytokine-mediated inflammatory responses[33]. The up-regulation of NF-kappaB observed in response to REV infection may provide a necessary signal required for enhanced virus entry and synthesis of viral proteins inside the cells[34, 35].

The discovery that differentially expressed genes of the major histocompatibility complex (MHC) play an important role in the immune response depended on the virus-host interaction[36]. In this study, we found that multiple immune function-related genes were up-regulated, such as MHC class I antigen, MHC BF1 class I, MHC BF2 class I, YFV, and the β_2_M gene. Beta-2-Microglobulin (β_2_M) contains a distinctive molecular structure called a constant-1 Ig superfamily domain that is shared with other adaptive immune molecules including MHC class I and class II[37]. The variation in MHC class I and β_2_M gene expression might provide insights into host-virus interactions, such as those involved with in vitro and in vivo infections of the Marek's disease virus[38]. Thus we propose that the complement system activated by REV infection might serve as a functional bridge between the innate and adaptive immune responses, and these correlation mechanisms allow an integrated host defence to pathogenic challenges[39].

### Mechanism of immunosuppression during the course of REV infection

Host immune organs can induce substantial damage by regulating the activity of tumour necrosis factor in the spleen due to host REV infection[1, 40]. When chickens are infected with REV, immunosuppression occurs early after infection and allows the viruses to reproduce gradually and cause disease, by inhibiting the immune responses of lymphocytes, monocytes, and macrophages[41–43]. Moreover, REV DNA has been found to be integrated at multiple sites in acutely infected chicken cells, resulting in cell apoptosis and substantial damage of the immune organs and inevitably leading to immunosuppression[44]. In vivo, expression levels of interferon (IFN)-alpha, IFN-beta, IFN-gamma, IL-1beta, IL-2, IL-3, IL-15, IL-17F, IL-18 and colony-stimulating factor (CSF)-1 are significantly down-regulated, whereas interleukin (IL)-4, IL-10, IL-13 and tumour necrosis factor (TNF)-alpha are markedly increased in PBMCs at all stages of infection. Thus, REV regulates host immune responses, thereby inhibiting T-cell proliferative responses[40].

As we know, virus infections induce a proinflammatory response including expression of chemokines and cytokines[45]. In this study, increased expression of *IL-6*, *CCL-20*, and *CCL-19* was observed after REV infection. Interleukin-6 (IL-6) is a potent pleiotropic cytokine that plays an important role in the immune response and regulates cell growth and differentiation[46, 47]. Dysregulated expression of IL-6 and its receptor are implicated in the pathogenesis of many diseases, including multiple immunorepressive diseases and cancers[48, 49]. IL-6 has been shown to be up-regulated during infections with other retroviruses[50] and avian immunosuppressive viruses[51–53].

SOCS-1 and SOCS-3 are members of the STAT-induced STAT inhibitor (SSI) family, also known as the suppressor of cytokine signalling (SOCS) family, that is involved in the inhibition of the JAK-STAT signalling pathway. SSI family members are cytokine-inducible negative regulators of cytokine signalling[54, 55]. SOCS-1 and SOCS-3 have been implicated in feedback effects on IL-6 signal transduction through binding to phosphorylated tyrosine residues of a component of its receptor (gp130)[56]. SOCS-1 can negatively regulate the TLR3-mediated innate immune response and inhibit the TLR-2- and TLR-4-mediated signalling pathways by targeting the adaptor protein Mal, which induces the ubiquitin-dependent pathway and is involved in signalling via TLR-2 and TLR-4[57, 58]. Additionally, SOCS-3 might inhibit the TLR3 signalling pathway through the ubiquitin-mediated degradation of TRAF-6[59], and it is widely accepted that the anti-inflammatory properties of SOCS-3 induce a reduction in TNF-alpha levels[60, 61]. Because SOCS-1 and SOCS-3 were significantly up-regulated in this study, the host response to REV infection suggests a balance between pro- and anti-inflammatory cytokines that may be critical for REV immunosuppression.

### Signalling pathways during the time course of REV infection

The systematic bioinformatics analysis of the differential expression of genes during the time course of REV infection showed that cytokine-cytokine receptor interactions, cell adhesion molecules, and the PPAR signalling pathway may be involved in mechanisms governing the interaction of REV and its host cell. This speculation may explain why chicken embryo fibroblasts infected in culture with REV caused a chronic infection with morphological transformation but without cell killing[62]; however, these steps require further testing and verification.

In our study, certain cytokine receptors such as *IL13RA2*, *IL22RA1*, and *STAT-1* were found to be involved in the JAK-STAT signalling pathway. Additionally, many studies have focused on the interferon (IFN)-regulated JAK-STAT pathway and understanding the mechanisms governing the transcription of interferon stimulated genes (ISGs)[27]. Therefore, we speculated that the regulation of the JAK-STAT signalling pathway participated in the pathogenesis of REV infection in a manner similar to that observed for ALV-J infection[15]. Several differentially expressed genes were involved in the MAPK and Wnt signalling pathways. A strong association of these signalling pathways with tumourigenesis has been reported by many studies[63–65], suggesting that they may be involved in tumourigenesis induced by REV.

## Conclusions

In conclusion, we identified a total of 1,785 differentially expressed genes and several immune pathways that are activated in response to REV infection of CEFs. While many of these genes and pathways have been previously associated with avian RNA virus infection, this study identified significant canonical pathways associated with REV infection. Additionally, we have provided further insight into the differences and similarities among differentially expressed genes implicate in inflammation, antiviral activity, and immunosuppression. It suggests that these strategies of host-adaptation by REV are involved in transcriptional control of immune responses. Further studies will be required to define the functions of the genes identified during in-vitro viral infections and to elucidate the virus and host mechanisms that modulate the host gene expression response.

## Supporting Information

S1 Table Selection of Genes showing over two fold alterations at all four time point. (XLS)

S2 Table List of transcripts in each cluster by hierarchical clustering and k-means clustering methods. (XLS)

S3 Table Significantly enriched Gene Ontology terms of the differentially expressed genes. (XLS)

S4 Table Profiling of differentially expressed genes in immune response term. (XLS)
